# Sim-to-Real for High-Resolution Optical Tactile Sensing: From Images to Three-Dimensional Contact Force Distributions

**DOI:** 10.1089/soro.2020.0213

**Published:** 2022-10-13

**Authors:** Carmelo Sferrazza, Raffaello D'Andrea

**Affiliations:** Institute for Dynamic Systems and Control, Department of Mechanical and Process Engineering, ETH Zurich, Zurich, Switzerland.

**Keywords:** tactile sensing, sim-to-real, machine learning, computer vision

## Abstract

The images captured by vision-based tactile sensors carry information about high-resolution tactile fields, such as the distribution of the contact forces applied to their soft sensing surface. However, extracting the information encoded in the images is challenging and often addressed with learning-based approaches, which generally require a large amount of training data. This article proposes a strategy to generate tactile images in simulation for a vision-based tactile sensor based on an internal camera that tracks the motion of spherical particles within a soft material. The deformation of the material is simulated in a finite element environment under a diverse set of contact conditions, and spherical particles are projected to a simulated image. Features extracted from the images are mapped to the three-dimensional contact force distribution, with the ground truth also obtained using finite-element simulations, with an artificial neural network that is therefore entirely trained on synthetic data avoiding the need for real-world data collection. The resulting model exhibits high accuracy when evaluated on real-world tactile images, is transferable across multiple tactile sensors without further training, and is suitable for efficient real-time inference.

## Introduction

Research on vision-based (or optical) tactile sensors aims to provide robots with high-resolution information about contact with external objects. However, while the images stemming from the various optical tactile sensing principles are intuitive and to some extent interpretable by human observations, the extraction of accurate physical quantities is challenging. In this regard, the complexity of mapping the information extracted from the images to the corresponding contact conditions mainly results from the fact that accurate modeling techniques for soft materials are generally not suitable for real-time applications. In addition, previous research has predominantly focused on the estimation of low-dimensional quantities (e.g., total contact force, center of contact), which may be sufficient for a limited range of tasks, but not for generic applications, as is the case for tasks that involve arbitrary points of contact.

The work discussed in this article targets both these topics, proposing a data-driven approach to reconstruct the three-dimensional (3D) distribution of the contact forces applied to the soft surface of a vision-based tactile sensor. The sensing strategy was presented in the authors' previous work^1^ and is based on the tracking of particles randomly spread within a soft gel. The use of data bypasses the need for modeling techniques with real-time guarantees, but as opposed to classical data-driven strategies, here the data necessary for training the learning architecture at the core of the method are entirely generated in simulation. Furthermore, the estimation of the contact force distribution directly yields both the total contact force (i.e., the component-wise integral of the force distribution) and the contact locations (i.e., the surface patches where the contact pressure is nonzero) and is additionally suitable to represent generic contact conditions with arbitrary points of contact, therefore providing high versatility across several tasks.

**FIG. 1.**
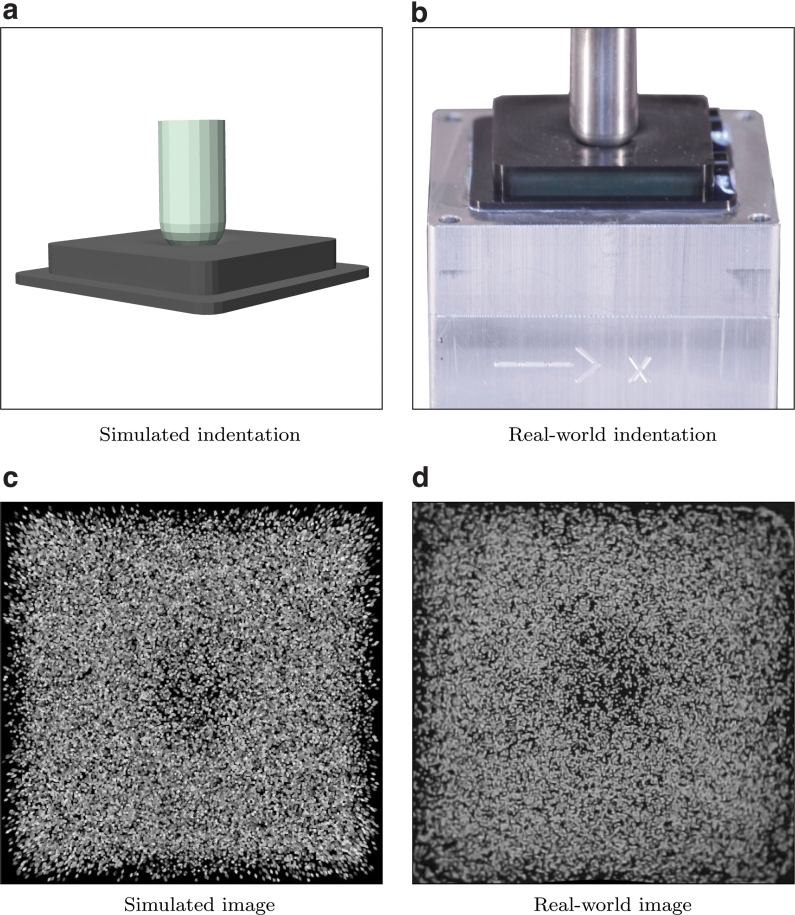
This work builds upon the generation of training images in simulation for a data-driven, vision-based tactile sensor based on the tracking of a spread of particles. **(c)** and **(d)** show two tactile images from simulation and reality, respectively, generated through the corresponding contact conditions shown in **(a)** and **(b)**. Color images are available online.

The main contributions of this work are the following:

- It details a method to simulate the images captured by a vision-based tactile sensor,^1^ starting from simulations based on the finite element method^2^ (FEM).
- It outlines two strategies to generate simulated datasets comprising tactile image features and labels. These strategies differ from the one presented in the authors' previous work,^3^ as they relax a small deformation assumption and simplify the transfer from simulation to reality. The datasets collected for this work comprise a variety of contact conditions, producing high shear and pressure forces with indenters of different shapes and sizes.
- It describes a tailored learning architecture, based on u-net,^4^ which can be trained entirely with simulated data obtained offline through high-fidelity FEM-based simulations. When evaluated on real-world tactile sensors, the architecture yields high accuracy in the reconstruction of the force distribution, achieving real-time inference up to a speed of 120 Hz (Fig. 1).

### Related work

In recent years, a number of tactile sensing principles^5^ have been developed to address the needs of the robotics community. Among these, vision-based tactile sensors^6^ use standard cameras^7^ or optical devices^8,9^ to infer the deformation of a soft membrane and obtain information about the contact with external objects that causes the deformation. This category of tactile sensors generally benefits from high resolution and ease of wiring, and its straightforward manufacture enables fast prototyping for robotic systems. Although the bulkiness of their sensing unit is the main limitation of such approaches, recent works have proposed compact solutions that exploit embedded cameras^10–14^ or mirrors.^15^

The sensory feedback provided by tactile sensors typically requires further processing, as it does not directly translate to the physical quantities of interest for robotic tasks. In this regard, model-based methods^16,17^ often rely on strong modeling assumptions (e.g., linear elasticity of the materials) to solve the processing task in an approximate manner, while data-driven methods^18–20^ aim to compute offline a mapping from raw data to the quantities of interest, to preserve accuracy while ensuring real-time inference.

While most of the literature has primarily focused on the estimation of low-dimensional physical quantities (e.g., total forces), recently several works have shifted the focus toward the estimation of distributed quantities, which aim to provide high-resolution tactile fields for a wide range of tasks. In the context of vision-based sensors, the estimation of the contact patches^17^ has been proposed, and the reconstruction of the contact force distribution has been discussed, both in a model-based^16^ and a data-driven^18^ manner. In addition, various approaches have been proposed outside the vision-based domain, with regard to the estimation of the deformation field^21^ and the pressure distribution.^22^

As a result of the possibility of collecting and generating accurate data offline, data-driven approaches generally exhibit smaller estimation errors than model-based methods.^8^ However, their bottleneck often lies in the fact that they require large amounts of training data, and they do not often generalize well when used in unseen contact conditions. To address the issue of data efficiency, a number of works have focused on generating training data in simulation to extract a model that retains its accuracy when used in the real world. Examples of such sim-to-real (or sim2real) transfers can be found in the literature for edge prediction^23^ and the estimation of the contact pressure^22^ and the deformation field.^24,25^ In previous work, a sim-to-real approach was presented to estimate the 3D force distribution^3^ for a limited range of scenarios.

This article presents two different methods to generate a dataset to train a data-driven approach entirely using FEM simulations, with the aim to reconstruct the 3D contact force distribution applied to a vision-based tactile sensor. Image features were extracted from the tactile images generated in simulation and mapped to three matrices representing the components of the force vectors applied over the soft sensing surface. The mapping was obtained using a tailored neural network architecture, which is able to capture various contact conditions as high shear and pressure forces, as well as indentations with flat or round objects. In addition, high accuracy was retained on real-world data, and the real-time speed could be more than doubled compared to previous work.^3^

### Outline

The sensing strategy and the hardware are described in the Hardware section, while the method to generate tactile images and extract the related features is presented in the Dataset Generation section. Starting from the generated dataset, the Learning Architecture section and the Results section describe the learning pipeline and the evaluation on simulated and real-world data, respectively. Final remarks and an outlook are included in the Conclusion section.

## Materials and Methods

### Hardware

The tactile sensor used in this work is based on a camera that tracks particles randomly distributed within a deformable material. The fabrication follows previous work^1^ and is detailed in Section 1 of the Supplementary Data. The sensing surface amounts to a rectangular prism of 32 × 32 × 6 mm. The soft materials have been characterized previously^18^ as hyperelastic materials following uniaxial, pure shear, and equibiaxial tension tests. The resulting second-order Ogden models^26^ were used for the FEM simulations discussed in the following sections.

### Dataset generation

Supervised learning is a natural data-driven way of processing sensory feedback and mapping raw data to the quantities of interest. In the context of vision-based tactile sensing, formulating the task in a supervised learning manner involves two crucial preliminary steps: (1) the choice of appropriate features to condense the information contained in the images; and (2) the formalization of finite-dimensional labels representing the quantities of interest. In addition, the availability of data necessary to train suitable learning architectures needs to be considered when addressing the formulation of the problem. In this work, training data were generated entirely in a finite element simulation environment with the objective of avoiding real-world data collection and maximizing the variability of the contact conditions without the need for complex hardware setups. A further advantage of collecting contact data in simulation is the possibility of extracting high-resolution tactile fields,^18^ which are otherwise not possible to measure with the commercially available commodity sensors. This work aimed to estimate the 3D force distribution, which is a condensed representation of several contact quantities. In fact, the force distribution encodes both the contact locations, which can be obtained by thresholding the normal component, and the total contact forces, which can be obtained by integrating the distribution over the sensing surface. As opposed to the deformation field, the contact patches are exactly encoded in the force distribution, while the deformation field can, for example, show deformation also where no contact is applied, as a result of the elasticity of the soft material. In addition, from the force distribution it is possible to compute the torques acting on the contact object, and all these properties remain valid for contact with multiple or arbitrary objects.

**FIG. 2.**
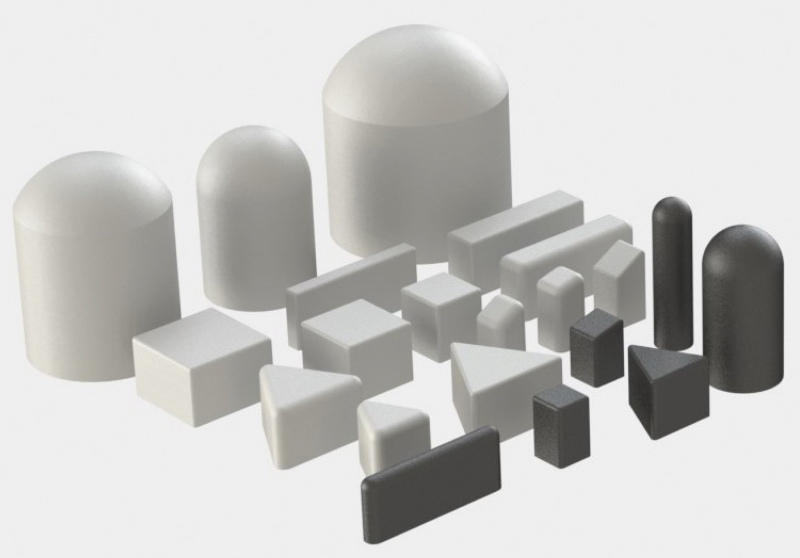
The figure shows the indenters used to collect the training data in the FEM simulations. Real-world realizations of the *black* indenters were used to collect the test data in reality. Note that the indentation surfaces correspond to the *top* surfaces in the figure. The *sharp* corners of the indenters were smoothed out to avoid a known singularity in the flat-punch indentation experiment.^36^ FEM, finite element method. Color images are available online.

An FEM simulation environment was created in Abaqus/Standard,^27^ details of this are provided in Section 2 of the Supplementary Data and in a previous work.^18^ Two training datasets were built by performing indentations in such an FEM environment with the 21 different indenters shown in Figure 2. The indentation trajectories were performed by either moving the indenter vertically and then purely horizontally or by prescribing indenter motions from different angles followed by random perturbations in the vicinity of the first indentation. A total of 3300 indentation trajectories (each comprising 50 indentation steps) were executed in simulation, with total forces up to 16 N in the vertical direction and up to 5 N in each of the horizontal directions. For each step of these trajectories, the contact force distribution and the displacement field were extracted at the nodes of a mesh refined around the contact between the indenter and the soft material. These quantities were further processed to compose two sets of features and labels, as described in the Training Features section for the displacement field and the Training Labels section for the force distribution. Since the training dataset was entirely generated in simulation, two test datasets were collected in reality as described in the Test Dataset section to verify the sim-to-real transfer and the real-world performance.

#### Training features

In this article, two different methods to extract image features are compared. The resulting types of features are denoted in the following as optical flow features and raw features, respectively. The starting points of both methods are the images captured by the internal camera, and for training purposes, these images were entirely generated in simulation. The soft materials were modeled in the FEM simulations as described in the Hardware section of this article and in Section 2 of the Supplementary Data. Highly accurate models were obtained for the same materials using state-of-the-art characterization experiments in previous work,^18^ where these models were also validated against a force-torque sensor. The Ogden model parameters used there were also employed in this work. A static friction coefficient of 0.9 was used, as it proved accurate for the indenters employed (see the experiments performed in Section 2 of the Supplementary Data).

**FIG. 3.**
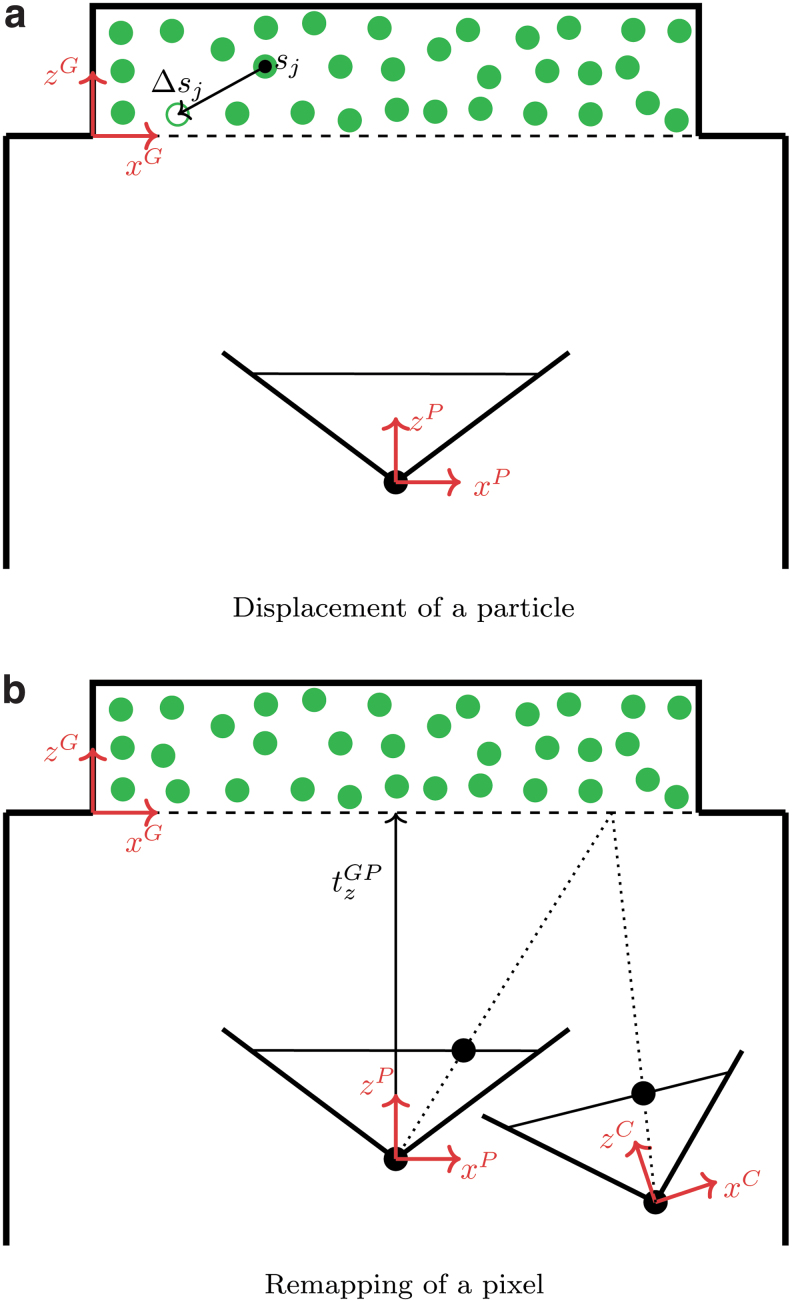
The drawings show the definition of the three coordinate systems used throughout the article: the gel coordinate system (*superscript G*), the pinhole camera coordinate system (*superscript P*), and the real-world camera coordinate system (*superscript C*). In **(a)**, an example of three-dimensional displacement of a particle originally placed at *s_j_* is depicted. In **(b)**, a pixel in the pinhole camera is mapped to the corresponding pixel in the real-world camera. Color images are available online.

A gel coordinate system (Fig. 3a) was defined by placing the origin at one of the bottom corners of the layer containing the particles, the *z* axis pointing toward the upper surface and the *x* and *y* axes aligned with two of the horizontal edges. For each indentation step performed in simulation, the FEM provides the displacement field of the soft layer that comprises the particles. This displacement field is provided at the discrete nodes of the FEM mesh. For such nodes, also the initial position (at rest, before deformation) is known. To generate the dataset, a random distribution of particles was sampled for each indentation step, and an inverse distance weighted scheme^28^ was used to interpolate the displacement field at the corresponding particle location $s_{j}^{G}$, for *j* = 0, …, *N*_p_ − 1, where *N*_p_ is the number of particles and the superscript *G* indicates the gel coordinate system. The 3D displacement of the *j*-th particle is denoted in the following as $\Delta s_{j}^{G}$. The strategy followed was to project the particles to the image plane using an ideal pinhole camera model^29^ and only account for the camera's nonidealities at a later stage,^3^ as described in the Test Dataset section. Therefore, as depicted in Figure 3, the position $s_{j}^{G}$ and the respective displacement $\Delta s_{j}^{G}$ were first transformed from the gel coordinate system to the 3D pinhole camera coordinate system (indicated by the superscript *P*) as

(1) $$s_{j}^{P}=R^{GP}s_{j}^{G}+t^{GP}$$

(2) $$\Delta s_{j}^{P}=R^{GP}{\Delta s}_{j}^{G}$$

where the rotation matrix *R^GP^* and the translation vector $t^{GP}:=\left(t_{x}^{GP},t_{y}^{GP},t_{z}^{GP}\right)$ are the pinhole camera's extrinsic parameters. These parameters could be chosen arbitrarily, but they were actually chosen to be close to the real-world camera's extrinsic parameters, as discussed in the Test Dataset section. The pinhole image resolution was arbitrarily set to be 440 × 440 pixels, and although the focal length could also be chosen arbitrarily in this step, to exactly capture the region where the particle layer (which has a square horizontal section of 30 × 30 mm) is visible, this was set for both the image coordinates as

(3) $$f:=\frac{440}{30}t_{z}^{GP}.$$

The projection of the spherical particle centered at $s_{p}^{P}$ using the pinhole camera model results in an ellipse on the image plane.^30^ The derivation of the center, the axis lengths, and the orientation of each ellipse can be found in Section 3 of the Supplementary Data. The ellipses can then be drawn using the drawing functionality of OpenCV.*

For each indentation step, one image at rest (projecting all the particles, i.e., by setting $s_{p}^{P}=s_{j}^{P}$ for the *j*-th particle) and one image after deformation (setting $s_{p}^{P}=s_{j}^{P}+\Delta s_{j}^{P}$ for the *j*-th particle) were generated. The images were initialized with black pixels, and each ellipse was drawn with a random RGB color to perturb the data with additional variability. The images were then converted to grayscale in a second step. An example of a simulated image is shown in Figure 1c. To further increase the training robustness, the number of the particles within the gel was slightly perturbed at each indentation step. Training features were then extracted from the images using two different methods:

1. Optical flow features: For each indentation step, the dense optical flow between the image at rest and the image after deformation was computed using an algorithm based on Dense Inverse Search.^31^ The per-pixel flow was then subsampled performing an average pooling in a grid of 88 × 88 bins. The two Cartesian components of the optical flow resulted in two matrices, which were concatenated into a two-channel matrix. This method differs from previous work,^3^ where optical flow features were directly computed from the FEM displacement field, assuming that the density of the particles remained constant during an indentation. In reality, this is not the case for large indentations, as the particles tend to spread radially under pressure and the method presented here can cope with such conditions.
2. Raw features: The two images for each indentation step were subsampled to 88 × 88 pixels and concatenated into a two-channel image, which was directly fed to the training algorithm.

#### Training labels

The same set of labels described in the following was assigned to each set of features to compose two separate training datasets. For each indentation step, the FEM simulations provide the 3D contact force distribution at the surface nodes of the FEM mesh. Dividing the surface into a grid of 20 × 20 bins,^18^ the force components at the nodes falling inside a bin were summed to obtain a 20 × 20 three-channel matrix, representing the training label for the corresponding indentation step datapoint. Examples of ground truth labels are shown in Figure 8. In this work, a node was assigned to a certain bin depending on its initial position before deformation, to simplify the binning at the boundaries of the gel, which can vary with deformation. As an alternative, it would also be possible to assign the nodes to the bins according to the position after deformation, by introducing an adaptive binning strategy at the boundaries of the grid.

#### Test dataset

To evaluate the real-world performance of the models described in the Learning Architecture section, 1100 test datapoints were collected in an experimental setup, using a programmable milling machine (Fehlmann PICOMAX 56 TOP) to make vertical and shear-dominant indentations with the six black indenters shown in Figure 2, as well as multicontact indentations with two spherically-ended indenters placed at different heights. The resulting test dataset induced total forces up to 4.5 N in the vertical direction and up to 3.8 N in each of the horizontal directions. These ranges differ from the training data ranges, which also included data inducing larger strains and where the material model fit was less accurate. Such higher strain data showed improved generalization in the learning and for this reason were included in the training dataset.

**FIG. 4.**
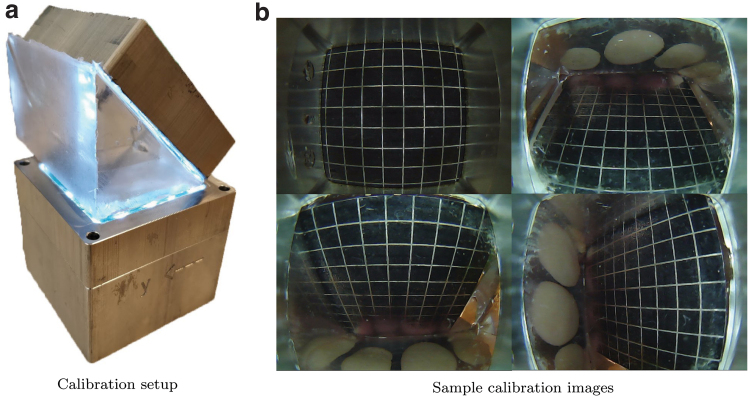
The calibration images, examples of which are shown in **(b)**, were shot through a silicone medium during fabrication, to account for the refraction index of the soft materials. As shown in **(a)**, this was done straight after casting the *first layer* (see Section 1 of the Supplementary Data), by placing additional silicone parts between the *first layer* and a grid pattern attached to an aluminum surface. Color images are available online.

During the test data collection procedure, the images taken by the real-world camera were recorded. Since the models were trained with features obtained from images generated using a pinhole camera projection, a further procedure was needed to account for the camera's nonidealities on real-world images.^3^ This procedure is denoted as remapping, and it essentially maps the pixels from a real-world image (converted to grayscale) to the pixels of an image of the same scene as if it was taken from the ideal pinhole camera used for the training dataset. The remapping procedure requires two main steps:

1. Calibration step: during fabrication, seven images of a grid pattern were shot through a silicone medium (Fig. 4). In this way, it is possible to account for the refractive index of the soft materials. Using a fisheye camera calibration toolbox,^32^ the images were used to obtain both the extrinsic parameters *R^GC^* and *t^GC^* of the real-world camera, as well as a transformation function from the actual camera 3D coordinate system to the real-world image. The extraction of the extrinsic parameters was achieved by providing the calibration toolbox with a calibration image where the origin of the grid pattern coincided with the origin of the gel coordinate system.
2. Interpolation step: for each pixel in the fictitious pinhole image, the corresponding pixel in the real-world image was obtained, using a procedure sketched in Figure 3b. For this step, the pixels were assumed to be placed approximately at a fixed *z* coordinate in the pinhole camera coordinate system, set here with the bottom of the gel layer. The details of the interpolation procedure are further detailed in Section 4 of the Supplementary Data.

As shown in Figure 3b, the approximation introduced above has a smaller effect when the pinhole extrinsic parameters *R^GP^* and *t^GP^* are close to the real-world camera extrinsic parameters *R^GC^* and *t^GC^*, respectively. As mentioned in the Training Features section, since the pinhole extrinsic parameters can be set arbitrarily, these were indeed chosen to be close to the expected real-world extrinsic parameters to limit the impact of the approximation. While the calibration parameters are fixed across images of the same camera and can be computed offline, the interpolation step needs to be performed for each image.

**FIG. 5.**
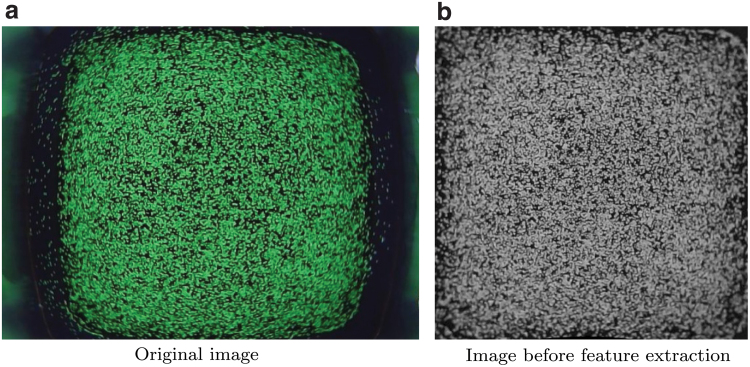
The original image taken from the real-world camera, shown in **(a)**, was converted to grayscale and remapped as if it was taken from the ideal pinhole camera. A refinement procedure was applied to account for inaccuracies introduced during calibration. The resulting image in **(b)** shows the particle layer in its actual squared geometry, covering the entire image frame. Color images are available online.

The extrinsic parameters obtained during calibration are very sensitive to the exact placement of the grid pattern for the corresponding calibration image. This requires pressing the grid pattern against the silicone medium just enough to remove the air in the middle without penetrating the soft material, which is challenging to achieve in reality. Therefore, a grid search (in the submillimeter range) was performed in the vicinity of the translation vector *t^GC^*, to make the particles in a sample remapped image taken at rest match the entire image frame. For this, after a series of dilation and erosion steps, a bounding box around the pixels can be easily computed using OpenCV and compared to the frame boundaries. A refined remapped image is shown in Figure 5, where lens distortion effects and misalignments were successfully compensated for.

After remapping, the same image features described in the Training Features section were extracted from the images. Since no real-world sensor can provide ground truth contact force distributions, these were extracted in simulation as described in the Training Labels section and in previous work^18^ and assigned to the corresponding features to compose two test datasets. Note that since the real-world camera nonidealities can be compensated in the remapping step, which does not affect training, this enables the transfer of models trained on the pinhole data across multiple instances of fabricated sensors, provided that the camera calibration is performed as described above. The remapping procedure described here aims to compensate only for the camera mismatches and does not serve as a calibration for the FEM model, which was independently characterized in previous work,^18^ as further detailed in the Supplementary Data.

### Learning architecture

**FIG. 6.**
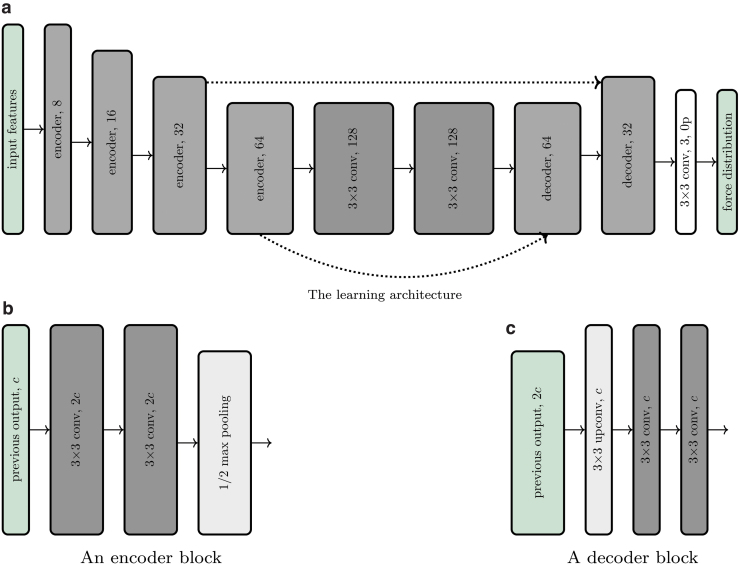
In **(a)**, a diagram of the learning architecture is shown. The encoder and decoder blocks are summarized in **(b, c)**, respectively. All the *blocks in green* serve as placeholders. “3 × 3 conv, *c*” indicates a convolutional layer with a 3 × 3 filter size and *c* output channels, while “3 × 3 upconv, *c*” indicates an upconvolution that doubles the input size. The *dotted lines* [omitted in **(b)** and **(c)**] indicate the concatenation of an earlier layer output with upsampled information. After each convolutional layer, with the exception of the *white* one before the final output, batch normalization and rectified linear units were used. “0p” indicates *no padding*. Where not indicated, all convolutional filters have unit zero padding and unit stride. Color images are available online.

The same learning architecture was used for both training datasets, that is, on those containing optical flow features and raw features, respectively. The architecture consists of a convolutional neural network, designed as a lightweight version of u-net,^4^ and tailored to the estimation of the force distribution from tactile features. In fact, this estimation problem can be formulated as an image-to-image translation^33^ (known also as pixel-wise regression). A sketch of the architecture is shown in Figure 6. The neural network exhibits an encoder-decoder structure, where feature information is first increased in the contraction step by doubling the channels between each pooling operation. In the decoding step, the force distribution is then computed through upconvolutions and concatenations of high-resolution features extracted during the contraction step. As a result, the architecture has the effect of both capturing context and enabling precise localization.

## Results

The learning architecture was trained twice from scratch using (1) the training dataset comprising averaged optical flow features and discretized force distribution labels and (2) the training dataset comprising raw image features and discretized force distribution labels. Both datasets were generated entirely in simulation, and both sets of training features contained two-channel 88 × 88 matrices (or images), as described in the Training Features section. The architecture was trained with the AdamW optimizer^34^ by minimizing a mean-squared loss (normalized by the maximum value per channel) with a learning rate of 1e-3 and a batch size of 256. During training, the datasets were randomly augmented by appropriately flipping the features and labels, exploiting the symmetry of the gel geometry and the pinhole camera projection. For the raw-feature dataset, the images were additionally augmented by perturbing the image brightness and adding salt-and-pepper noise.

After training in PyTorch,^†^ the models were converted to the ONNX format and used in real-time through the ONNX Runtime framework.^‡^ This generally led to a 4 × inference speedup on the CPU of a standard laptop (dual-core, 2.80 GHz), compared to the inference in PyTorch.

The performance of both trained models was evaluated on the corresponding synthetic validation datasets, picked randomly as the 20% of the indentation trajectories in the appropriate training dataset. In addition, the models were evaluated on the corresponding real-world test dataset described in the Test Dataset section. Table 1 summarizes the results based on two different error metrics for each force component: (1) *RMSE*, that is, the root-mean-squared error on the respective component of the force distribution, and (2) *RMSET*, that is, the root-mean-squared error on the respective component of the total force, which was obtained by summing the force distribution over all the bins. The range of total forces in the corresponding dataset is also shown in the table. In addition, Table 2 reports the mean and the standard deviation of the absolute errors, for the bin-wise and total force predictions on the real-world test data.

**Table 1.** The Table Shows the Error Metrics of the Trained Models on the Validation Datasets Extracted in Simulation and the Test Datasets Collected in Reality, for Both the Cases Where Optical Flow Features and Raw Features Were Used as Inputs

	RMSE [*N*]			RMSET [*N*]			Range of total forces [*N*]		
	x	y	z	x	y	z	x	y	z
Optical-flow (sim)	0.006	0.006	0.013	0.187	0.164	0.577	−5.0–5.0	−5.0–5.0	−16.0–0
Raw-feature (sim)	0.006	0.005	0.012	0.120	0.132	0.314	−5.0–5.0	−5.0–5.0	−16.0–0
Optical-flow (real)	0.006	0.007	0.018	0.190	0.230	0.914	−3.2–3.2	−3.8–3.8	−4.5–0
Raw-feature (real)	0.005	0.007	0.014	0.267	0.296	0.362	−3.2–3.2	−3.8–3.8	−4.5–0

*RMSE*, root-mean-squared error on the respective component of the force distribution; *RMSET*, root-mean-squared error on the respective component of the total force.

**Table 2.** The Table Shows Additional Error Metrics on the Real-World Test Sets in Terms of the Absolute Errors for Bin-Wise and Total Force Predictions, Namely the Mean Absolute Error and the Standard Deviation of the Absolute Errors

	MAE [*N*]			SDAE [*N*]		
	x	y	z	x	y	z
Optical-flow (bin)	0.001	0.001	0.004	0.006	0.007	0.018
Raw-feature (bin)	0.001	0.001	0.003	0.005	0.007	0.014
Optical-flow (total)	0.103	0.110	0.645	0.159	0.202	0.648
Raw-feature (total)	0.099	0.107	0.238	0.248	0.275	0.273

*MAE*, mean absolute error; *SDAE*, standard deviation of the absolute errors.

As the numerical results indicate, there is a slight difference in accuracy between the horizontal and vertical components of the predictions. This may be explained by the fact that during the vertical indentations, the shear forces were rather small or close to zero. More importantly, the raw-feature model outperformed the optical-flow model in most of the metrics on the corresponding real-world test dataset. In fact, while in practice the location accuracy for both models was similar, the optical-flow features tend to mitigate the differences across indenters under real-world noise, therefore resulting in inaccurate force predictions. In contrast, overall the raw-feature model showed a better transfer from simulation to reality, especially retaining a considerably higher accuracy in the vertical component. In addition to the difference in accuracy, the model trained on the raw image features does not require the extraction of the optical flow, which was the bottleneck for the model trained on optical flow features. As a result, since the model inference only takes about two milliseconds, the whole raw-feature pipeline (including the image acquisition and remapping) runs in real-time at 120 Hz on CPU, compared to the 50 Hz of the optical-flow pipeline.

**FIG. 7.**
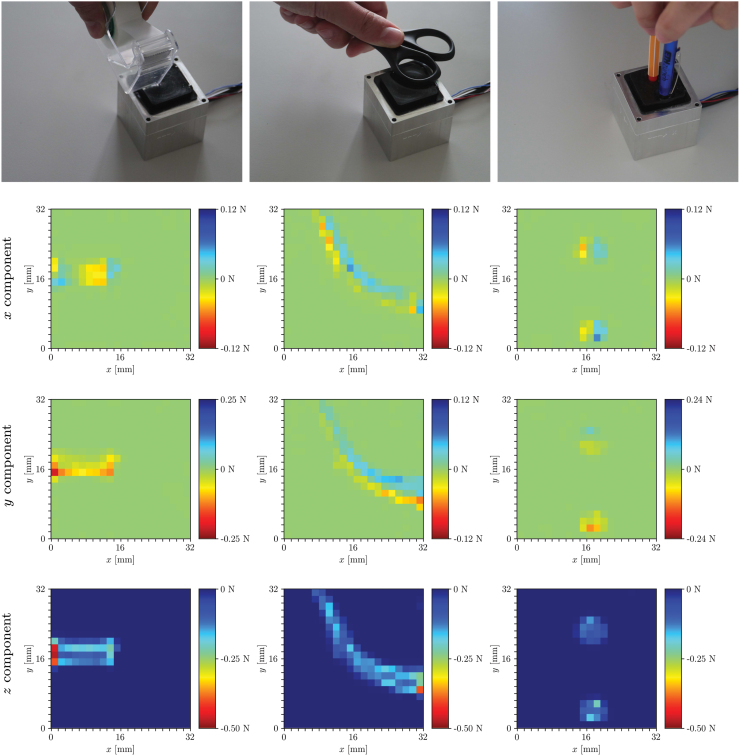
The figures show sensible predictions for the different contact conditions that are shown in the first row. The *x*, *y*, and *z* components of the predicted force distributions are shown in the *second*, *third*, and *fourth rows*, respectively. In the *first column*, the tape dispenser was initially pressed against the gel and then translated to induce higher shear forces in the negative *y* direction. In the second column, the contact with an object that differs significantly from those in the training set is shown, while the *third column* shows the contact with multiple bodies (not included in the training data), the lower of which is laterally translated as shown by the asymmetrical shear component in the *y* direction. Color images are available online.

**FIG. 8.**
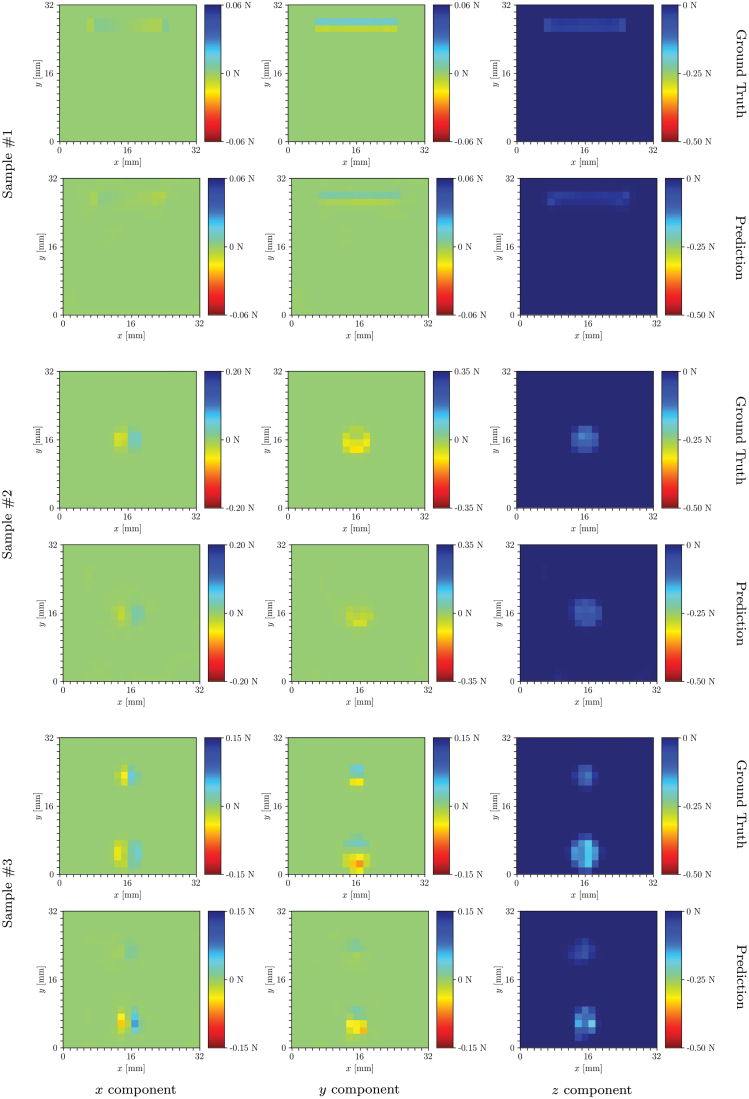
The figures show the ground truth (*first*, *third*, and *fifth rows*) and predicted (*second*, *fourth*, and *sixth rows*) force distribution components (*x* in the first column, *y* in the second column, and *z* in the third column) for different samples and indenters in the real-world test dataset. Predictions were made with the raw-feature model. The *first two rows* show vertical indentation; the *third* and *fourth rows* show a shear-dominant indentation, and the *last two rows* a multicontact indentation. Color images are available online.

The real-time performance of the raw-feature pipeline is shown in the Supplementary Video S1, where contact conditions with arbitrary objects were explored (Fig. 7). The estimation of the force distributions on samples in the test set with the model trained on raw features is shown in Figure 8. Additional results and comparisons are available in Section 5 of the Supplementary Data.

## Conclusion

This work has discussed strategies to simulate the images captured by a vision-based tactile sensor. Starting from FEM simulations, the displacement field was processed to generate training features for a supervised learning architecture that mapped these features to contact force distribution labels. The resulting models are directly transferable across multiple instances of real-world sensors, since the training procedure does not make use of real-world images. Two different strategies were compared, with the model obtained from raw features outperforming a model based on optical flow features for both real-world accuracy and inference speed. In addition to providing a physical quantity directly interpretable across robotic tasks, the extraction of accurate force distributions also provides an abstraction from the image pixels that bypasses the remaining mismatch between real and simulated images.

Since this work aimed to provide a comparison between the two approaches, the same input and output sizes were used for both strategies. However, given the gain in prediction speed, the raw-feature approach may be extended to use higher resolution features or to predict the force distribution on a finer grid by trading off the sensing frequency. As shown in Table 1, a gap still remains between simulation and reality, which could be addressed by explicitly addressing the domain transfer problem. This issue will be the subject of future work.

The simulator described in this work provides highly accurate force distribution labels to train learning-based models suitable for real-time inference. However, the simulator itself is not running in real time, due to the computational complexity of the FEM. While this was not in the scope of this work, different simulation techniques can trade off accuracy to achieve real-time capabilities and become suitable to warm-start the training of tactile policies in simulation, as detailed in a related work.^35^

## Supplementary Material

Supplemental data

Supplemental data

## Notes

^*^

https://opencv.org/

^†^

https://pytorch.org/

^‡^

https://www.onnxruntime.ai/
