# Supplementary material for: Sim-to-Real for High-Resolution Optical Tactile Sensing: From Images to Three-Dimensional Contact Force Distributions
Source: Soft Robot. 2022 Oct 13;9(5):926–37. doi: 10.1089/soro.2020.0213 (PMC9595648; doi:10.1089/soro.2020.0213)
Supplement: Supplemental data [file Supp_DataS1.pdf]

Sim-to-real for high-resolution optical tactile sensing: From images to 3D contact force distributions - Supplementary Material

Carmelo Sferrazza and Raffaello D'Andrea *

1. Fabrication

The soft materials are arranged in three layers, as shown in Fig. 1(a), and were poured (after degassing in a vacuum chamber) on top of the camera (ELP USBFHD06H with a fisheye lens) and the surrounding LEDs with the mold lying on one side, i.e., with the camera pointing sideways, as shown in Fig. 1(b). The fabrication strategy followed previous work,[1] but it is presented extensively here to provide additional details for reproducibility:

1. A first layer of Elastosil® RT 601 RTV-2 (mixing ratio 7:1, shore hardness 45A) was poured into the mold, closed with a first lid (see Fig. 1(c)). The mold was then placed into an oven at 80 °C for 20 minutes for curing. This layer serves as a stiff base and facilitates light diffusion.

2. A release agent (Mann Ease Release™ 200) was sprayed before assembling the second lid, shown in Fig. 1(d). Then, a layer of Ecoflex™ GEL (mixing ratio 1:1, very soft, with shore hardness 000-35), mixed with green, fluorescent spherical particles (with a diameter of 150 to 180 μm), was poured into the mold. The mold was finally placed into an oven at 80 °C for 20 minutes for curing.

3. Finally, a layer of Elastosil® RT 601 RTV-2 (mixing ratio 25:1, shore hardness 10A), mixed with black silicone color (Elastosil® Color Paste FL), was poured into the mold, closed with a third lid, shown in Fig. 1(e). The mold was then placed into an oven at 80 °C for 45 minutes for curing. This layer is stiffer than the Ecoflex GEL, and shields the sensor from damage and light disturbances.

4. After removing the last lid, the sensor was placed back in the oven at 60 °C for 8 hours. This step has been shown to reduce stiffening caused by the aging of the materials.[2]

The two soft upper layers amount to a rectangular prism of 32×32×6 mm.

2. The FEM simulation environment

The FEM simulation environment was created in Abaqus/Standard[3] following previous work[2], where the Ecoflex GEL and the black Elastosil layer have both been characterized as hyperelastic materials using second-order Ogden models[4]. Given the large difference in hardness, the stiff base layer was considered rigid[2] in the FEM simulations discussed in the article. The contact between the top surface and the indenters was modeled as a hard contact and discretized with a surface-to-surface method. The basic Coulomb friction model available in Abaqus was employed, where the friction coefficient was assumed to be constant and was used as a tuning parameter, as described in the following.

*Institute for Dynamic Systems and Control, ETH Zurich, 8092 Zurich, Switzerland.
Correspondence to: csferrazza@ethz.ch

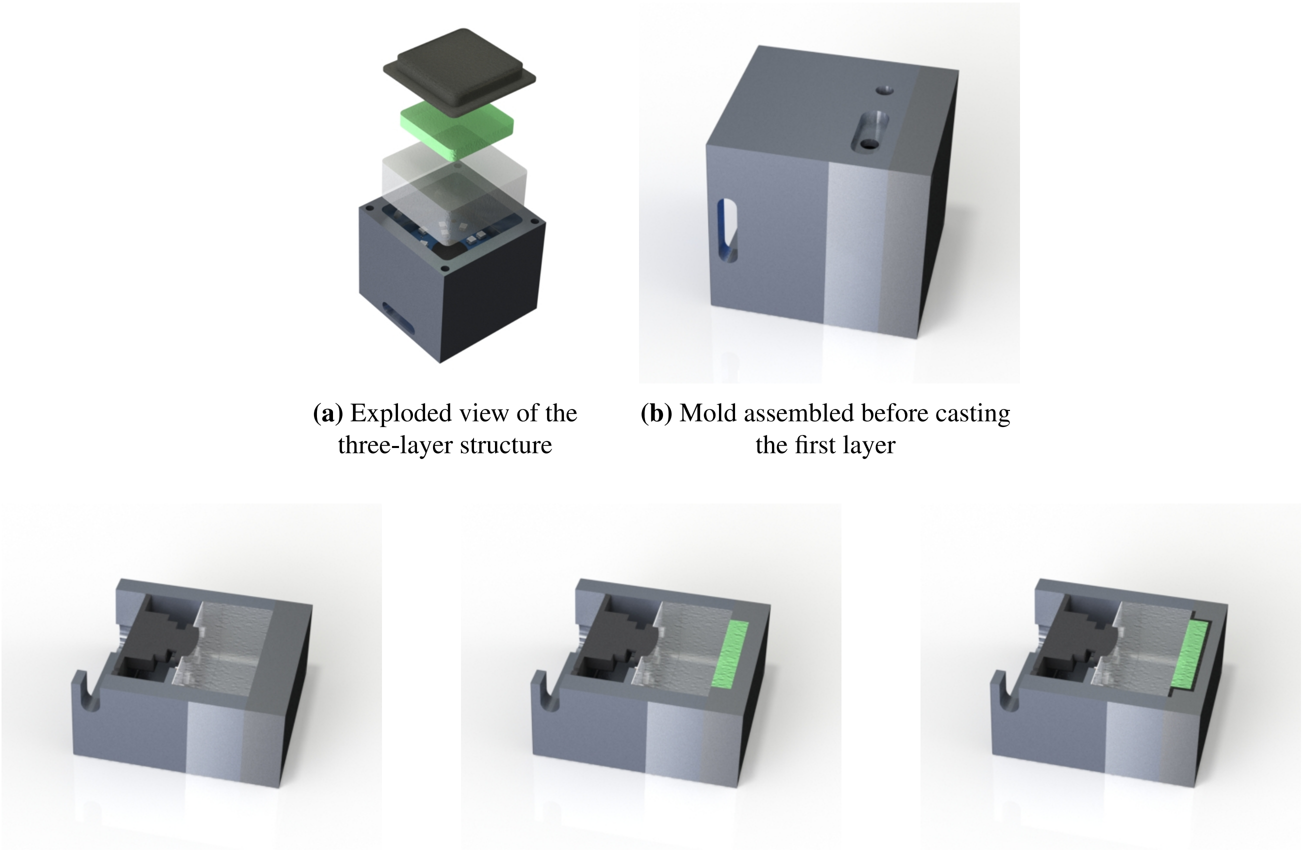

(a) Exploded view of the three-layer structure

(b) Mold assembled before casting the first layer

(c) Cross-section after casting the first layer

(d) Cross-section after casting the second layer

(e) Cross-section after casting the third layer

Figure 1. The figure details the sensor's fabrication. The soft materials are arranged in a three-layer structure (see (a)) on top of the camera and the LEDs, and were poured into the mold from the side, through lateral cavities such as those shown in (b). Three different lids (see (c)-(e)) were employed for each of the soft layers.

The material characterization followed state-of-the-art techniques based on uniaxial tension, pure shear, and equibiaxial tension tests. The models obtained required no further calibration for the simulations described in this article. In fact, the characterization tests were entirely independent of the evaluation experiments carried out in this work. In order to verify the consistency of the Ogden models and the related FEM simulations, the previous work[2] also showed an accurate total force agreement when the same vertical indentation experiments were performed both in the FEM environment and the real world, where the total force was obtained from the readings of a commercial six-axis F/T sensor. Such verification experiments were augmented here to test the total force accuracy also in the case of shear-predominant or multi-contact indentations. The results of these experiments are shown in Fig. 2, where the total force resulting from FEM indentations was compared with the force measured by an F/T sensor (ATI Mini27 Titanium with an horizontal resolution of 0.03 N and a vertical resolution of 0.06 N) when repeating the same indentations in the real-world. The real-world experiments were carried out by mounting the F/T sensor and the appropriate indenters to the spindle of a controllable milling machine. The friction coefficient was tuned to a value of 0.9 using a single 3D-printed rough indenter in the experiment shown in (a). However, the remaining experiments showed generalization to different indentation shapes (see (b)) and a limited loss of accuracy for indenters of a different and smoother material, such as stainless steel (see (c)). In addition, the friction coefficient has a limited influence on the accuracy of the vertical component of the total force, as shown for the multi-contact experiment performed in (d) with stainless steel indenters.

The indentation trajectories collected in the FEM environment, which were employed to generate training data, were randomized as follows:

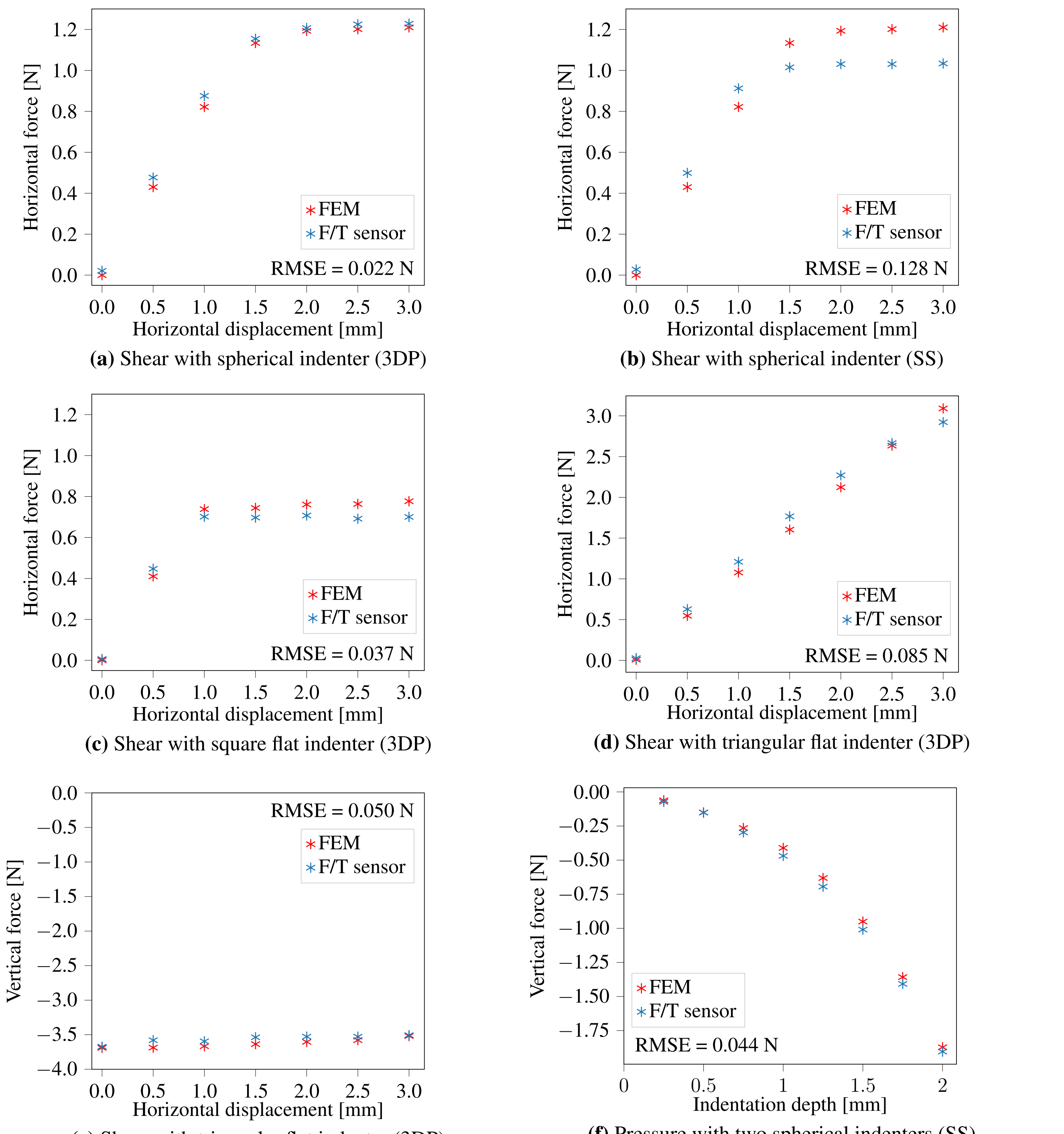

(a) Shear with spherical indenter (3DP)

(b) Shear with spherical indenter (SS)

(c) Shear with square flat indenter (3DP)

(d) Shear with triangular flat indenter (3DP)

(e) Shear with triangular flat indenter (3DP)

(f) Pressure with two spherical indenters (SS)

Figure 2. The plots show the agreement (measured by the root-mean-square error, RMSE) between the total force resulting from FEM indentations (in red) and the readings of an F/T sensor (in blue) when repeating the same indentations in a controlled experimental scenario. While the FEM simulations are performed assuming a fixed friction coefficient, the real-world experiments employed indenters that were either 3D-printed (3DP) or made of stainless steel (SS). In (a), a 3DP spherically-ended indenter was first pressed 2 mm down in the center of the sensing surface. Then it was laterally sheared in one direction, and the horizontal force was recorded at discrete steps. In (b), the same experiment was repeated with an SS indenter with the same geometry. In (c), the shear experiment was carried out with a square flat-ended indenter at a depth of 1 mm, and in (d) with a triangular flat-ended indenter at a depth of 2 mm. The corresponding vertical force for the same experiment as in (d) is shown in (e), where a slight decrease in force was detected during shear from both the FEM simulation and the F/T sensor. In (f), two SS spherically-ended indenters (both attached to the same F/T sensor in the real world) were employed to make vertical indentations on the sensing surface, with the total vertical force recorded at discrete steps. Since the two indenters had a constant difference in height of 1.1 mm, the first four steps (up to 1 mm depth) resulted from contact with only one of the indenters, while the remaining steps (after 1 mm) resulted from a double indentation.

1. First, for each trajectory, one of the 21 indenters modeled in simulation was selected randomly and translated to a random horizontal position over the sensing surface with a randomized orientation.

2. Then, 80% of the time, a vertical indentation followed by randomized horizontal translations was simulated. In the remaining 20% of the time, random 3D displacements were directly simulated in the vicinity of the initial position. Such a split in the training trajectories aimed to favor the typical robotic manipulation case, where shear motion happens after a vertical grasp. Each indentation trajectory was split into 50 steps, with the maximum intra-step displacement constrained to 0.1 mm to facilitate convergence. The maximum depth reached by the indenters was 2 mm, while the maximum lateral displacement from the start of the indentation was 3 mm. Static steps were employed, therefore neglecting time-dependent material effects, which are however limited, as shown in Fig. 5.

3. Projection of a particle onto the image plane

The projection of the spherical particle centered at $s_{\mathrm{p}}^P := \left(x_{\mathrm{p}}^P, y_{\mathrm{p}}^P, z_{\mathrm{p}}^P\right)$ via the pinhole camera model results in an ellipse on the image plane,[5] see Fig. 3(a). The pixel length r of the major axis of each ellipse can be computed via the projection formulas in the plane containing the camera's optical axis and the camera ray passing through the center of the spherical particle. An example projection in this plane is shown in Fig. 3(b). The coordinate $\tilde{x}_{\mathrm{p}}^P$ can be computed from the horizontal coordinates of the center of the particle as,

$$\tilde{x}_{\mathrm{p}}^P = \sqrt{\left(x_{\mathrm{p}}^P\right)^2 + \left(y_{\mathrm{p}}^P\right)^2}. \tag{1}$$

Then, from the figure, it follows that:

$$\alpha = \arctan\left(\frac{z_{\mathrm{p}}^P}{\tilde{x}_{\mathrm{p}}^P}\right), \tag{2}$$

$$\beta = \arcsin\left(\frac{R}{\sqrt{\left(\tilde{x}_{\mathrm{p}}^P\right)^2 + \left(z_{\mathrm{p}}^P\right)^2}}\right), \tag{3}$$

$$\gamma = \alpha - \beta, \tag{4}$$

where R is the radius of the sphere. The pixel length r of the major axis can then be computed as:

$$\tilde{x}_{\mathrm{r}}^P = \tilde{x}_{\mathrm{p}}^P + R\sin\gamma, \tag{5}$$

$$z_{\mathrm{r}}^P = z_{\mathrm{p}}^P - R\cos\gamma, \tag{6}$$

$$\tilde{x}_{\mathrm{l}}^P = \tilde{x}_{\mathrm{p}}^P - R\sin(\gamma + 2\beta), \tag{7}$$

$$z_{\mathrm{l}}^P = z_{\mathrm{p}}^P + R\cos(\gamma + 2\beta), \tag{8}$$

$$r = \left| f\left(\frac{\tilde{x}_{\mathrm{r}}^P}{z_{\mathrm{r}}^P} - \frac{\tilde{x}_{\mathrm{l}}^P}{z_{\mathrm{l}}^P}\right)\right|. \tag{9}$$

The orientation of the ellipse on the image plane is fully determined by the horizontal position of the particle, and can therefore be computed trivially as:

$$\omega = \operatorname{arctan2}\left(y_{\mathrm{p}}^P, x_{\mathrm{p}}^P\right). \tag{10}$$

Additionally, the center of the ellipse can be computed by observing that $(u_{\mathrm{r}}, v_{\mathrm{r}})$ and $(u_{\mathrm{l}}, v_{\mathrm{l}})$ correspond to the projection of $(\tilde{x}_{\mathrm{r}}^P, z_{\mathrm{r}}^P)$ and $(\tilde{x}_{\mathrm{l}}^P, z_{\mathrm{l}}^P)$, respectively, onto the image plane:

$$x_{\mathrm{r}}^P = \tilde{x}_{\mathrm{r}}^P\cos\omega, \quad y_{\mathrm{r}}^P = \tilde{x}_{\mathrm{r}}^P\sin\omega, \tag{11}$$

$$u_{\mathrm{r}} = f\frac{x_{\mathrm{r}}^P}{z_{\mathrm{r}}^P} + u_0, \quad v_{\mathrm{r}} = f\frac{y_{\mathrm{r}}^P}{z_{\mathrm{r}}^P} + v_0, \tag{12}$$

$$x_{\mathrm{l}}^P = \tilde{x}_{\mathrm{l}}^P\cos\omega, \quad y_{\mathrm{l}}^P = \tilde{x}_{\mathrm{l}}^P\sin\omega, \tag{13}$$

$$u_{\mathrm{l}} = f\frac{x_{\mathrm{l}}^P}{z_{\mathrm{l}}^P} + u_0, \quad v_{\mathrm{l}} = f\frac{y_{\mathrm{l}}^P}{z_{\mathrm{l}}^P} + v_0, \tag{14}$$

where (u_0, v_0) are the coordinates of the pinhole image center.

Therefore, the pixel coordinates of the center of the ellipse are:

$$u_{\mathrm{p}} = \frac{u_{\mathrm{r}} + u_{\mathrm{l}}}{2}, \tag{15}$$

$$v_{\mathrm{p}} = \frac{v_{\mathrm{r}} + v_{\mathrm{l}}}{2}. \tag{16}$$

Finally, noting that the pixel length of the minor axis of the ellipses does not vary with the horizontal coordinates of the sphere,[5] this length can be computed for a trivial case, that is, when the center of a particle lies on the optical axis (i.e., $x_{\mathrm{p}}^P = y_{\mathrm{p}}^P = 0$). The same formulas as in (1)-(9) can be employed, since for this special case the projection results in a circle, where both the major axis and the minor axis of the ellipse correspond to the diameter. Using the center, the axis

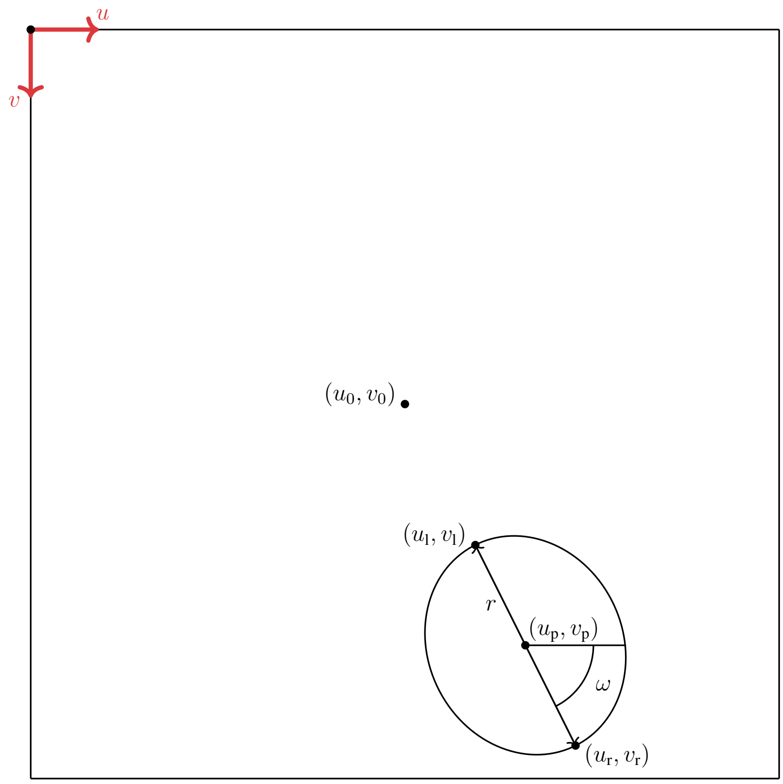

(a) Projection of a sphere onto the image plane

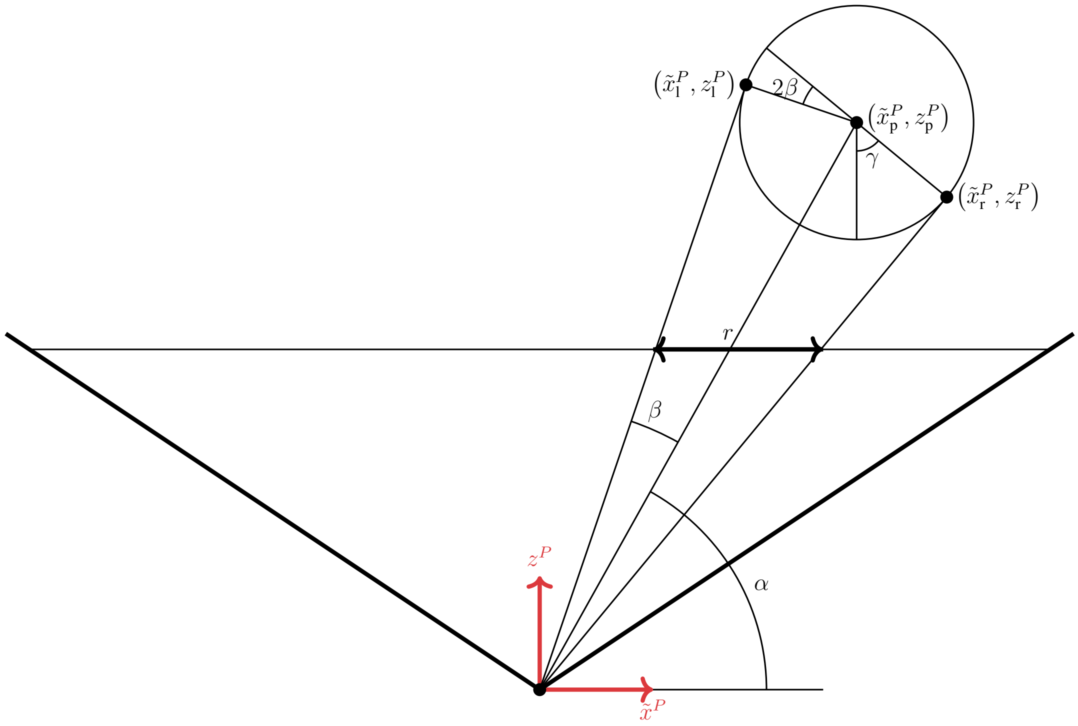

(b) 2D view of the projection of a sphere

Figure 3. The figures show that the projection of a sphere corresponds to an ellipse (see (a)) in the image plane. The length r of the major axis of this ellipse can be computed via 2D geometry in the plane that contains the optical axis and the ray passing through the camera and the center of the sphere.

lengths and the orientation of each ellipse, these can be drawn using the drawing functionality of OpenCV*.

4. Remapping

As shown in Fig. 4, for a pixel $p := (u, v)$ in the image plane of the pinhole camera, a 3D point $s^P := (x^P, y^P, t_z^{GP})$ was retrieved using the pinhole projection equations as:

$$x^P = \frac{t_z^{GP}}{f}(u - u_0), \quad (17)$$

$$y^P = \frac{t_z^{GP}}{f}(v - v_0). \quad (18)$$

The 3D point was then converted to the coordinate system of the real-world camera, indicated with the superscript C, through the corresponding rotation and translation operations:

$$s^C = R^{GC} \left(R^{GP}\right)^{-1} \left(s^P - t^{GP}\right) + t^{GC}. \quad (19)$$

The corresponding pixel in the real-world image was then retrieved via the transformation function obtained from the calibration toolbox.

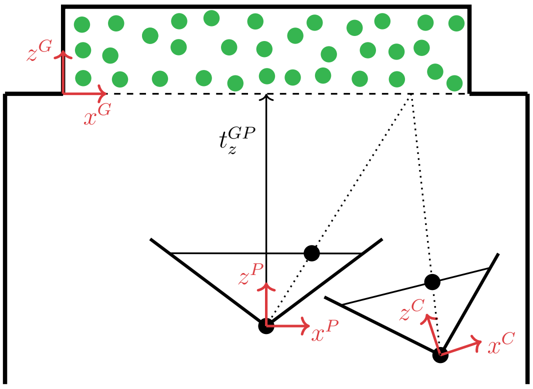

Figure 4. In the figure, a pixel in the pinhole camera is mapped to the corresponding pixel in the real-world camera.

5. Results

This section presents supplementary results and illustrations in addition to those in the main article. Fig. 6 compares optical flow examples obtained for the same indentation in simulation and reality. In Fig. 5, a programmable milling machine was employed to make indentations using two of the test indenters, and the total force recorded with an F/T sensor was compared with the real-time prediction of the neural network presented in the main article.

Table 1 and Table 2 show in detail the different error metrics listed by data subgroups for the real-world test dataset, depending on the type of indentation or the indenter employed. Both tables are based on metrics computed using the raw-feature model described in the main article. It can be noted that shear-dominant indentations may present a decrease in accuracy in the prediction of the z component of the force. This is partly due to the fact that shear-dominant data generally showed higher noise, as a small misalignment in the indenter mounting may lead to considerably different behavior of the material during the shearing trajectory. In addition, as shown in the main article, the model tended to generalize to multi-contact indentations. However, the performance for such contact conditions may be further improved by including a portion of multi-contact data in the training dataset. Among the six indenters employed, the results generally showed a correlation between the range of forces and the errors recorded. In addition, it turned out to be very challenging to accurately align the tilted-plane indenter (which is the indenter that shows a deeper side in Fig. 2 of the main article) with the reference system of the gel for data collection. For this reason, shear data were not collected with such an indenter.

Furthermore, Fig. 7 shows that a diverse dataset is crucial for generalization. The samples in the figure correspond to the first two in Fig. 8 of the main article, but the predictions were made with the network trained in previous work.[6] This network was only trained with vertical indentations made with a spherically-ended indenter. To evaluate the generalization, the first two rows show a vertical indentation made with a cylindrical indenter, while the third and fourth rows show a shear-dominant indentation made with a spherically-ended indenter. While the network in previous work[6] showed sensible predictions for some indenters different from the one used for training, the figure shows how, in contrast, the

*https://opencv.org/

		Vertical	Shear-dominant	Multi-contact
RMSE	x	0.003	0.010	0.004
	y	0.005	0.011	0.004
	z	0.013	0.018	0.013
RMSET	x	0.034	0.548	0.088
	y	0.057	0.606	0.061
	z	0.277	0.465	0.488
MAE (bin)	x	0.001	0.002	0.001
	y	0.001	0.002	0.001
	z	0.002	0.004	0.002
MAE (total)	x	0.022	0.333	0.060
	y	0.035	0.342	0.046
	z	0.168	0.368	0.345
SDAE (bin)	x	0.003	0.010	0.004
	y	0.004	0.011	0.004
	z	0.012	0.017	0.012
SDAE (total)	x	0.025	0.436	0.065
	y	0.045	0.500	0.040
	z	0.221	0.284	0.345
Range (total)	x	-0.2–0.2	-3.2–3.2	-0.3–0.3
	y	-0.6–0.6	-3.8–3.8	-0.3–0.3
	z	-4.5–0	-3.8–0	-3.5–0

Table 1. The table reports in detail the different error metrics (using a raw-feature model) for each of the three types of real-world indentations, that is, vertical, shear-dominant, and multi-contact indentations. The abbreviations are defined as in the main article. The Newton unit was omitted here for all the values.

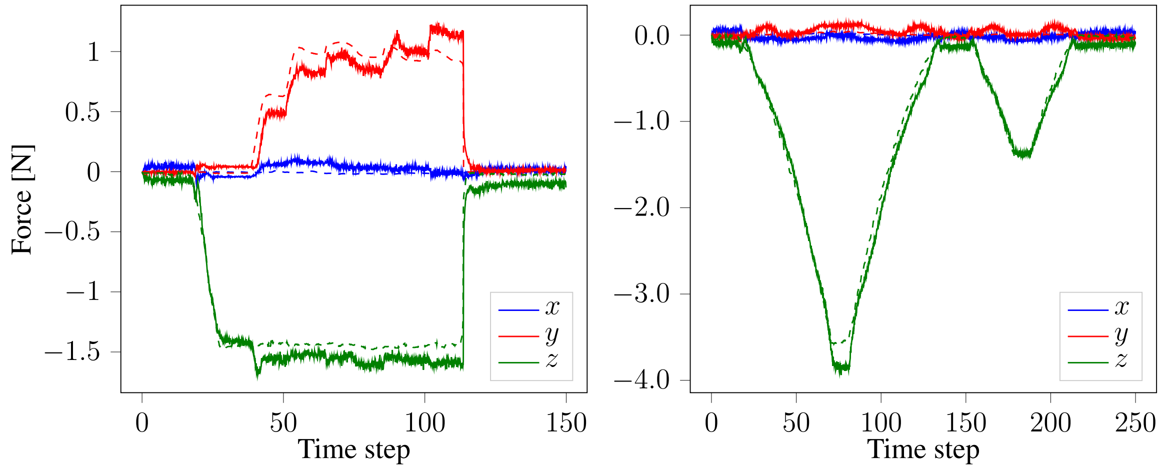

(a) Shear with cylindrical indenter

(b) Pressure with triangular flat indenter

Figure 5. The plots compare the total force computed from the predictions of the neural network (solid lines) against the readings of an F/T sensor (dashed lines) for each of the three force components. In (a), a thin cylindrical indenter was first pressed against the sensing surface up to a depth of 1 mm, then it was laterally displaced in the y direction in discrete steps up to 3 mm, and finally lifted. In (b), two pressure cycles were executed with a triangular flat indenter, first up to 2 mm, then up to 1 mm. Although the network was only trained on static data, the predictions accurately capture the force trends in both the figures. The main inaccuracies can be observed for considerably larger deformations (where the material characterization in previous work[2] showed larger variance) with the triangular flat indenter, or during the unloading phase (where the material showed mild relaxation effects).

		Spherical large	Triangular	Square	Cylindrical	Spherical small	Tilted-plane
RMSE	x	0.003	0.009	0.007	0.007	0.004	0.002
	y	0.003	0.008	0.007	0.014	0.003	0.002
	z	0.008	0.022	0.015	0.021	0.008	0.014
RMSET	x	0.194	0.411	0.306	0.494	0.082	0.034
	y	0.129	0.449	0.293	0.644	0.027	0.047
	z	0.154	0.478	0.231	0.498	0.082	0.664
MAE (bin)	x	0.001	0.002	0.001	0.001	0.001	0.001
	y	0.001	0.002	0.001	0.003	0.001	0.001
	z	0.001	0.005	0.003	0.005	0.001	0.003
SDAE (bin)	x	0.004	0.009	0.007	0.007	0.003	0.002
	y	0.003	0.008	0.007	0.014	0.003	0.002
	z	0.007	0.021	0.014	0.020	0.008	0.013
MAE (total)	x	0.080	0.197	0.128	0.219	0.030	0.023
	y	0.057	0.208	0.142	0.336	0.021	0.037
	z	0.111	0.379	0.185	0.395	0.058	0.596
SDAE (total)	x	0.177	0.361	0.278	0.443	0.076	0.025
	y	0.116	0.398	0.257	0.550	0.017	0.030
	z	0.106	0.291	0.138	0.302	0.059	0.293
Range (total)	x	-1.2–1.2	-3.2–3.2	-2.2–2.2	-3.2–3.2	-0.1–0.1	-0.01–0.01
	y	-1.2–1.2	-3.2–3.2	-2.2–2.2	-3.8–3.8	-0.1–0.1	-0.01–0.01
	z	-1.7–0	-4.3–0	-2.0–0	-4.6–0	-1.0–0	-1.3–0

Table 2. The table reports in detail the different error metrics (using a raw-feature model) for each of the six real-world indenters employed to collect the test dataset (and shown in the main article). The Newton unit was omitted here for all the values.

network does not generalize well to light pressure conditions with the cylindrical indenter. In particular, the network predicted the typical force profile for a spherically-ended indenter. In addition, the shear-dominant indentation was also mispredicted, with the y component of the force distribution predicted as symmetrical, which is the typical force profile in a vertical indentation.

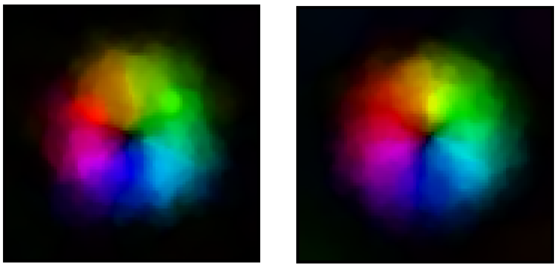

(a) Simulation **(b)** Reality

Figure 6. Comparison of the optical flow obtained in simulation (a) versus reality (b). The color represents the direction, while darker regions represent smaller displacements.

References

[1] C. Sferrazza and R. D'Andrea, "Design, Motivation and Evaluation of a Full-Resolution Optical Tactile Sensor," *Sensors*, vol. 19, no. 4:928, 2019.

[2] C. Sferrazza, A. Wahlsten, C. Trueeb, and R. D'Andrea, "Ground Truth Force Distribution for Learning-Based Tactile Sensing: A Finite Element Approach," *IEEE Access*, vol. 7, pp. 173438–173449, 2019.

[3] Dassault Systèmes, *Abaqus/Standard User's Manual, Version 2019*, 2019.

[4] R. W. Ogden, "Large deformation isotropic elasticity – on the correlation of theory and experiment for incompressible rubberlike solids," in *Proceedings of the Royal Society of London. A. Mathematical, Physical and Engineering Sciences*, vol. 326, pp. 565–584, 1972.

[5] D. S. Wokes and P. L. Palmer, "Perspective Projection Of A Spheroid Onto An Image Plane," *SIAM Journal on Imaging Sciences*, 2008.

[6] C. Sferrazza, T. Bi, and R. D'Andrea, "Learning the sense of touch in simulation: a sim-to-real strategy for vision-based tactile sensing," in *Proceedings of the IEEE International Conference on Intelligent Robots and Systems*, 2020.

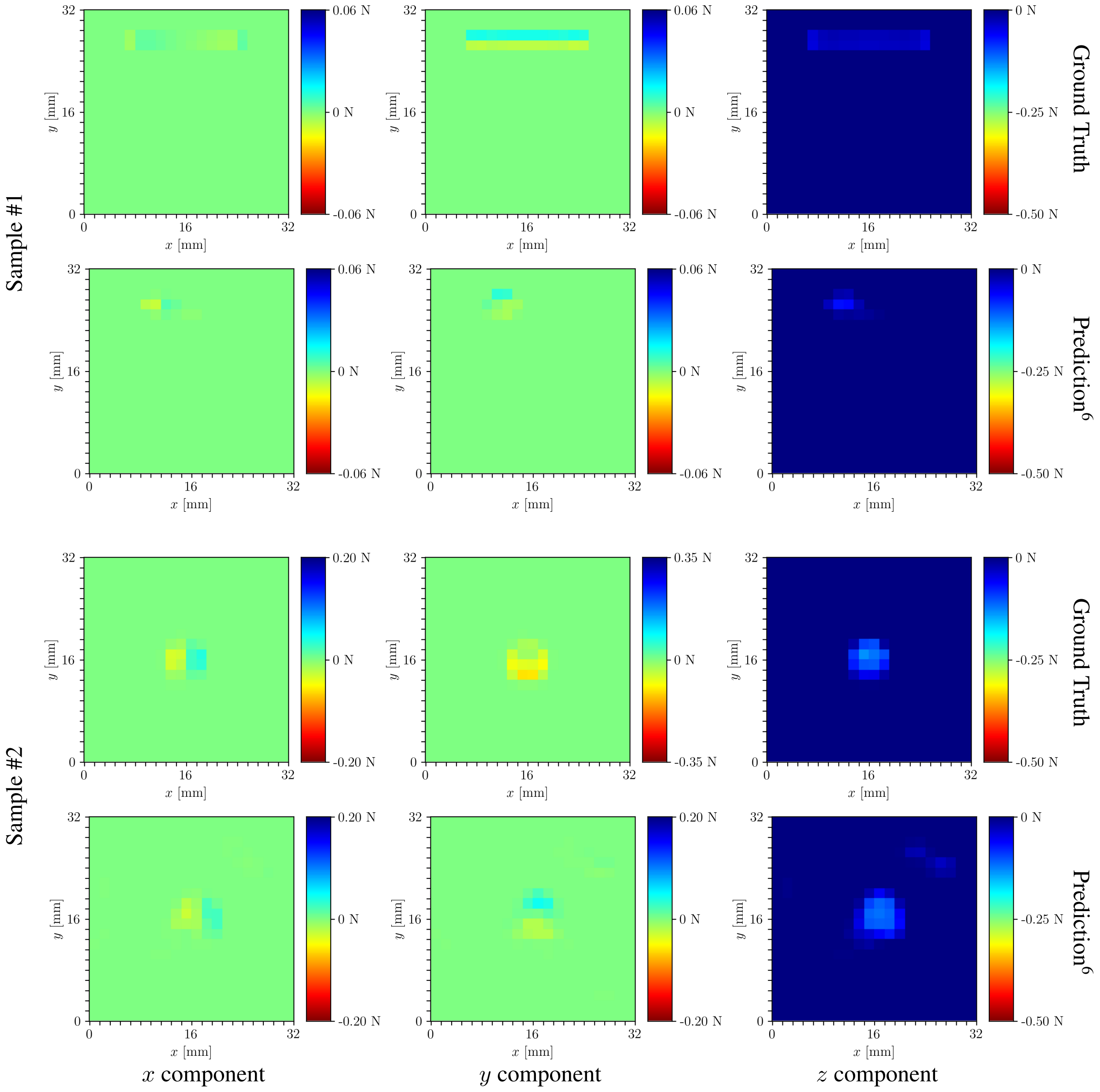

Figure 7. The figures show the ground truth (first and third rows) and predicted (second and fourth rows) force distribution components (x in the first column, y in the second column, and z in the third column) for the first two samples shown in Fig. 8 in the main article, collected with two different indenters in the real world. Predictions were made with the model trained in previous work[6], where only vertical indentations made with a spherically-ended indenter were contained in the training dataset. The first two rows show a vertical indentation with a cylindrical indenter; the third and fourth rows show a shear-dominant indentation with a spherically-ended indenter. Note how the model trained in previous work[6] does not generalize well to such cases.
